# *Laurus nobilis*: Composition of Essential Oil and Its Biological Activities

**DOI:** 10.3390/molecules22060930

**Published:** 2017-06-03

**Authors:** Lucia Caputo, Filomena Nazzaro, Lucéia Fatima Souza, Luigi Aliberti, Laura De Martino, Florinda Fratianni, Raffaele Coppola, Vincenzo De Feo

**Affiliations:** 1 Department of Pharmacy, University of Salerno, Via Giovanni Paolo II, 132, 84084 Fisciano (Salerno), Italy; lcaputo@unisa.it (L.C.); luceia.souza@ufrgs.br (L.F.S.); luigialiberti83@libero.it (L.A.); ldemartino@unisa.it (L.D.M.); 2 Istituto di Scienze dell’Alimentazione, Consiglio Nazionale delle Ricerche (ISA-CNR), Via Roma 64, 83100 Avellino, Italy; mena@isa.cnr.it (F.N.); fratianni@isa.cnr.it (F.F.); 3 Post-Doctoral by National Counsel of Technological and Scientific Development (CNPq/Brazil), 70000-000 Brasília, Brazil; 4 Dipartimento di Agricoltura, Ambiente e Alimenti-Università del Molise, Via de Sanctis snc, 86100 Campobasso, Italy; coppola@unimol.it

**Keywords:** *Laurus nobilis* L., essential oil, antibacterial activity, antifungal activity, cytotoxicity, adenylate cyclase 1 (ADCY1), Central Nervous System (CNS)

## Abstract

*Laurus nobilis* is native to the southern Mediterranean region and cultivated mainly in Europe and the USA as an ornamental and medicinal plant. The chemical composition of the essential oil (EO) from leaves of *L. nobilis,* collected in Southern Italy, was studied by GC and GC-MS. In all, 55 compounds were identified, accounting for 91.6% of the total essential oil. 1,8-Cineole (31.9%), sabinene (12.2%), and linalool (10.2%) were the main components. Antimicrobial and antifungal activities of EO and 1,8-cineole were determined in vitro. The cytotoxicity of the EO was evaluated against SH-SY5Y cell line, as well as the influence of the EO on the expression of adenylate cyclase 1 (ADCY1), suggesting possible essential oil effects on the Central Nervous System.

## 1. Introduction

The laurel, *Laurus nobilis* L., an evergreen tree or shrub belonging to the family Lauraceae, is native to the south parts of Europe and the Mediterranean area; this plant is widely cultivated in many countries of this region. Its dried leaves and the essential oil (EO) deriving from leaves are used as a valuable spice and flavoring agent in culinary and food industry. The leaves have been used, in Iranian folk medicine, to treat epilepsy, neuralgia, and parkinsonism [1,2]. Leaves and fruits have been reported to possess aromatic, stimulant, and narcotic properties [3]. Several studies reported the antimicrobial and the antioxidant properties of laurel essential oil and/or extracts [4,5,6]. The leaves of *L. nobilis* are traditionally used orally to treat the symptoms of gastrointestinal problems, such as epigastric bloating and flatulence [7]. The essential oil of laurel leaves is widely used in the perfume and soap industries [8]. Moreover, it has been used for relieving hemorrhoid and rheumatic pains [2]. It also has diuretic and antifungal activities [1,2,9].

The present study describes the composition of the essential of leaves of *L. nobilis* and some of its biological activities. In particular, we evaluated the possible antimicrobial effects against different strains of bacteria and fungi, its cytotoxicity on SH-SY5Y cells and its possible subcellular effects in SH-SY5Y cells are used to evaluate the possible effects on Central Nervous System (CNS).

## 2. Results

### 2.1. Essential Oil Yield and Composition

The hydrodistillation of the leaves of *L. nobilis*, harvested in Montecorice (Campania, Southern Italy) provided an essential oil characterized by a typical odor, in a yield of 0.57% of the yield is calculate on the fresh weight. Table 1 reports the chemical composition of the oil; the compounds are listed according to their elution on an HP-5 MS capillary column.

In all, 55 compounds were identified, accounting for 91.6% of the total oil. Oxygenated monoterpenes represent 48.6% of the EO, with 1,8-cineole (31.9%), sabinene (12.2%), and linalool (10.2%) being the main components. Other components were α-terpinyl acetate (5.9%), α-pinene (5.8%), α-terpineol (3.3%), methyl-eugenol (3.3%), neoiso-isopulegol (2.5%), eugenol (1.6%), β-pinene (1.4%), and γ-terpinene (1.0%). Sesquiterpenes represent 3.4% of the oil, the hydrocarbons 3.2% (β-funebrerne 0.5%, β-elemene 0.4%, spathulenol 0.4%), and the oxygenated compounds 0.2%.

### 2.2. Antimicrobial Activity

The antibacterial activity of the essential oil and of 1,8-cineole was tested, at different amounts, on five bacterial strains, belonging to both Gram-positive and Gram-negative bacteria—namely *Staphylococcus aureus*, *Bacillus cereus* (4313), and *B. cereus* (4384) representative of the Gram-positive; and *Escherichia coli* and *Pseudomonas aeruginosa* characteristics Gram-negative bacteria. The results of the activity, namely the inhibition of the diameters calculated as mm, are given in Table 2.

The essential oil showed significant antimicrobial activity against all microorganisms as early as the lesser amount used in the test (0.4 µL), giving inhibition zones between 6.33 and 8.66 mm. Such results were particularly appealing, as far as we are concerned, considering the zone of inhibition produced by tetracycline. In fact, the diameters of inhibition produced by 0.4 µL of the essential oil resulted in two cases almost equal (versus *E. coli*) or even superior (versus *S. aureus*) to that produced by tetracycline. It should be also emphasized that 1 and 2 µL of essential oil resulted in a sensitivity by all the tester strains superior to that given by their contact with tetracycline, giving zones of inhibition between 13.33 and 18 mm. *B. cereus* 4313 turned out the most sensitive organism among those tested, and it was also more sensitive if compared the other strain of *B. cereus* used in the assay.

In all cases, the essential oil was more effective than 1,8-cineole, which was ineffective against *E. coli* (except at 2 µL/mL) and against *P. aeruginosa* and *S. aureus* at concentration of 0.4 µL/mL.

The results of halo inhibition test were confirmed by MIC test (Table 3). In fact, except on *Bacillus cereus* 4313, whose growth resulted inhibited using the same volume of EO and 1,8-cineole, in all the other cases, the essential oil was able to inhibit the growth of the microbial strains with a lower volume than 1,8-cineole.

### 2.3. Antifungal Activity

In Table 4 we report the widths (mm) of the inhibition halos exhibited by different volumes of *L. nobilis* EO and of 1,8-cineole against different moulds (*Aspergillus niger*, *A. versicolor*, *Penicillium citrinum* and *P. expansum*). Overall, fungal strains were sensitive both to the *L. nobilis* EO and 1,8-cineole. *P. expansum* exhibited the most sensitivity to the action of the essential oil, just at the minimal volume used in the test, giving halos of 8 mm. *A. versicolor* was more resistant only at the lower volume used.

### 2.4. Cytotoxicity of 1,8-cineole and Laurus nobilis Essential oil

**Figure 1:**
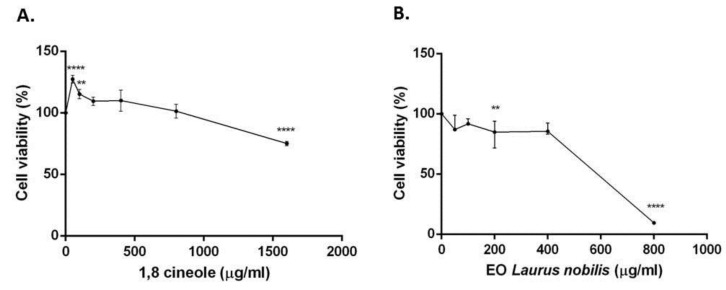
Cell viability calculated as percentage after MTT assay. Cells were treated with different concentrations (1600–50 µg/mL) of 1,8-cineole (**A**) and *L. nobilis* essential oil (**B**), for 24 h and solvent (DMSO, 0.1%) alone. Data are the mean ± SD of three experiments ** *p* < 0.01, **** *p* < 0.0001 vs. DMSO.

The treatment of SH-SY5Y neuroblastoma cells with (1600–50 µg/mL) of 1,8-cineole and *Laurus nobilis* essential oil for 24 h resulted in a low cytotoxic activity. 1,8-Cineole and essential oil showed an IC_50_ > 2000 g/mL and IC_50_ = 47,106 µg/mL, respectively. However, the treatment with essential oil resulted in a stronger cytotoxicity (IC_50_ < 500 µg/mL) (Figure 1).

### 2.5. Adenylate Cyclase (ADCY1): Western Blot Analysis

**Figure 2:**
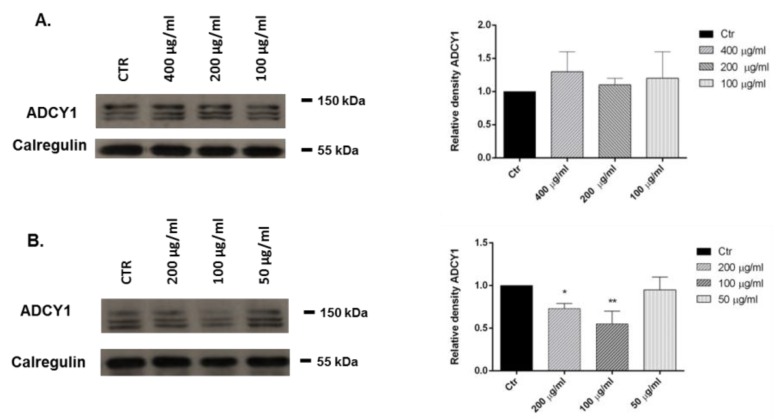
Relative expression levels of the ADCY1 protein in SH-SY5Y cells treated with 1,8-cineole (**A**) and *Laurus nobilis* essential oil (**B**). Each panel shows the densitometry of bands in the treated groups and control. Values are the mean ± SD in each group (*n* = 3). * *p* < 0.05, compared to control (ANOVA followed by Dunnett’s multiple comparison test).

We investigated the effects of 1,8-cineole and *L. nobilis* essential oil in SH-SY5Y human neuroblastoma cells. Representative Western blots and quantitative densitometry for adenylate cyclase 1 (ADCY1) protein expression in SH-SY5Y following exposure to different concentrations of 1,8-cineole and the essential oil are shown in Figure 2. Treatments of SH-SY5Y neuroblastoma cells with 1,8-cineole had no influence on ADCY1 expression (Panel A), whereas a treatment with 200 and 100 µg/mL of *L. nobilis* essential oil for 24 h significantly reduced ADCY1 expression (Panel B).

## 3. Discussion

In the composition of the essential oil of *L. nobilis*, 1,8-cineole, sabinene, and linalool are the main components, with other compounds being present in low percentages or even in traces.

The comparison with most recent literature concerning the chemical composition of the essential oil of *L. nobilis* from other Mediterranean areas, showed substantial differences: 1,8-cineole percentage found (31.9%) is lower than the values recorded in other studies: Turkey 44.97% [10], Tunisia 56.0% [11], Cyprus 58.59% [12], Morocco 52.43% [5], but similar to Algerian essential oil (34.62%) [13]. In our sample, the amount of sabinene was 12.2%, higher than in the essential oils analyzed by Derwich and coworkers [5], Snuossi and co-workers [11], and Yalcin [12] who found percentages of sabinene of 6.13, 3.5, and 6.13%, respectively. Linalool was found in concentrations comparable to previous studies [10,13] but in essential oil analyzed by Derwick and coworkers [5] linalool was not found.

The inhibition halo technique is often used to assess antibacterial activity of vegetal extracts and essential oils [14]. Our results on the antimicrobial activity confirm that the behavior of an antimicrobial agent can be not only species-specific but also strain-specific [15,16]. The available literature reports several studies ascertaining the antibacterial activity of the essential oil of *L. nobilis* against different pathogens [6,17,18,19]. The Algerian laurel essential oil versus *E. coli* and *Ps. aeruginosa* produced an inhibition halo of 13.73 mm and 10.73, respectively. The oil produced an inhibition halo of 13.03 mm when assayed versus *S. aureus* [19]. In our experiments, the antimicrobial activity resulted higher than that of the essential oil of *L. nobilis* collected in Turkey, if tested against *E. coli* O157:H7 [20]. Considering the chemical composition of the essential oil, we could hypothesize a synergistic action at least of the main compounds present in, specifically monoterpenes and oxygenate monoterpenes, which have per se low antimicrobial activity when used as single compounds [21]. Other terpenes—such as α-pinene, β-pinene, γ-terpinene δ-3-carene, (+)-sabinene, and α-terpinene—showed a very low or no antimicrobial activity against 25 genera of bacteria [22]. These in vitro tests confirm that terpenes show ineffective antimicrobial activity when used as single compounds [23]. The *L. nobilis* essential oil showed different antifungal activity (Table 4) respect to previous studies: in fact, our results indicate a marked antifungal activity, superior, for example, respect to that observed by Simic and co-workers both against *Aspergillus* and *Penicillium* spp., in spite of its high content of 1,8-cineole [24]. Therefore, the diameter of the halo produced by 1,8-cineole against the fungi was less evident. For this reason, we hypothesize a synergistic effect among the different components of the *L. nobilis* EO, which, in a certain sense, cancel out the weakness demonstrated by the individual components. The remarkable effectiveness exhibited by the studied essential oil may be interesting for example in the manufacturing and food processing, which, as is known, is increasingly directed to the use of green technologies able to safeguard the quality food, the environment, and especially the health of the consumer [25]. Therefore, the comforting results obtained using the essential oil of *L. nobilis* indicate that such oil could be used as an antimicrobial agent, also for health and food purposes. The use of this essential oil prolonged the shelf life of modified atmosphere-packed (MAP) fish fillets [26]. The laurel essential oil was also used to safeguard the quality and safety of fresh sausages, prolonging their shelf life too and also avoiding the problems related to fat oxidation [27].

The cytotoxic activity of the 1,8-cineole and the essential oil were evaluated in human neuroblastoma cell line (SH-SY5Y). The IC_50_ values were > 400 µg/mL, indicating that the substances were not cytotoxic, as judged by the criterion set by the National Cancer Institute, that stated that only natural substances with IC_50_ < 20 µg/mL were considered to be cytotoxic against the treated cells [28]. However, comparing the IC_50_ values, our findings indicated that *L. nobilis* EO is more cytotoxic than 1,8-cineole. This cytotoxicity can be probably attributed to a synergistic activity of this and other components.

Specific induction of apoptosis by 1,8-cineole was observed in human leukemia Molt 4B and HL-60 cells, but not in human stomach cancer KATO III cells [29]. However, this monoterpene was not cytotoxic against the human cancer in vitro models in the present study. Our results showed that the essential oil showed less cytotoxicity than the one tested on ACHN and C32 cell lines (IC50 202.62 and 209.69 µg/mL for ACHN and C32, respectively) [30] and the leaf extract tested on human neuroblastoma cell lines SK-N-BE(2)-C and SH-SY5Y [31].

Essential oils have been used for centuries as a traditional medicine, and currently used worldwide in the management of depression, anxiety, and stress-related disorders but there is very little verified science behind their use [32]. To clarify the traditional belief in the antiepileptic effects, and the use in neuralgia and parkinsonism of *L. nobilis* in folk medicine [1], we investigated the possible influence of the essential oil and its major components on ADCY1 expression. Our results showed that the treatment with 200 and 100 µg/mL of *L. nobilis* essential oil reduced ADCY1 expression in SH-SY5Y cell and, consequently, the intracellular production of cAMP. 1,8-Cineole had no effect on ADCY1 expression but we hypothesized that the essential oil effect is due to the presence of linalool (10.2%). Elisabetsky and coworkers showed that linalool possesses dose-dependent sedative effects in the Central Nervous System [33]. In our previous study, we reported that linalool is a component of many essential oils that can influence the expression of ADCY1 and ERK [34].

Moreover, in the complex phytochemical composition of the essential oil, one or more components might act synergistically to influence ADCY1 expression in SH-SY5Y cells.

## 4. Materials and Methods

### 4.1. Plant Material

*L. nobilis* leaves were collected in February 2016, in Montecorice, Cilento area (Campania, Southern Italy, 40°14′9″24 N, 14°59′13″20 E), 90 m above sea level. Representative homogeneous samples of population were collected during the balsamic time. The plant was identified by Prof. V. De Feo, on the basis of *Flora d’Italia* [35] and a voucher specimen has been deposited in the Herbarium of the Medical Botany Chair of the University of Salerno.

### 4.2. Isolation of Volatile Oil

One hundred grams of dried leaves of *L. nobilis* were ground in a Waring blender and then subjected to hydrodistillation for 3 h according to the standard procedure described in the European Pharmacopoeia [36]. The yellow essential oil was solubilized in *n*-hexane, filtered over anhydrous sodium sulphate, and stored under N_2_ at +4 °C in the dark, until tested and analyzed.

### 4.3. GC-FID Analysis

Analytical gas chromatography was carried out on a Perkin-Elmer Sigma-115 gas chromatograph (Pelkin-Elmer, Waltham, MA, USA) equipped with a FID and a data handling processor. The separation was achieved using a HP-5MS fused-silica capillary column (30 m × 0.25 mm i.d., 0.25 μm film thickness). Column temperature: 40 °C, with 5 min initial hold, and then to 270 °C at 2 °C/min, 270 °C (20 min); injection mode splitless (1 μL of a 1:1000 *n*-hexane solution). Injector and detector temperatures were 250 °C and 290 °C, respectively. Analysis was also run by using a fused silica HP Innowax polyethylenglycol capillary column (50 m × 0.20 mm i.d., 0.25 µm film thickness). In both cases, helium was used as carrier gas (1.0 mL/min).

### 4.4. GC/MS Analysis

Analysis was performed on an Agilent 6850 Ser. II apparatus (Agilent Technologies, Inc., Santa Clara, CA, USA), fitted with a fused silica DB-5 capillary column (30 m × 0.25 mm i.d., 0.33 μm film thickness), coupled to an Agilent Mass Selective Detector MSD 5973; ionization energy voltage 70 eV; electron multiplier voltage energy 2000 V. Mass spectra were scanned in the range 40–500 amu, with scan time of 5 scans/s. Gas chromatographic conditions were as reported in the previous paragraph; transfer line temperature, 295 °C.

### 4.5. Identification of Essential Oil Components

The identification of the essential oil constituents was based on the comparison of their Kovats retention indices (RIs), determined relative to the tR values of *n*-alkanes (C_10_–C_35_) with either those of the literature [37,38,39] and mass spectra on both columns with those of authentic compounds available in our laboratories by means NIST 02 and Wiley 275 mass spectral libraries [40]. The components’ relative concentrations were obtained by peak area normalization. No response factors were calculated.

### 4.6. Antimicrobial Activity

The inhibition halo test was performed in order to evaluate the potential antimicrobial activity of the *L. nobilis* essential oil and 1,8-cineole [15]. The Gram-negative *Escherichia coli* DMS 8579 and *Pseudomonas aeruginosa* ATCC 50071, and the Gram positive *Bacillus cereus* DSM 4313, *Bacillus cereus* 4384 and *Staphylococcus aureus* DSM 25693 were the bacterial strains tested in this study. Bacteria were purchased from the Deutsche Sammlung von Mikroorganismen und Zellkulturen GmbH (DSMZ). Nutrient broth (Sigma Aldrich, Milano, Italy) was used as medium of bacterial growth at 37 °C for 18 h. The optical densities of all cultures were adjusted to match a 0.5 McFarland standard of 1 × 10^8^ colony-forming units (CFU)/mL and spread onto Nutrient agar plates. The essential oil of *L. nobilis* as well as the pure component 1,8-cineole were re-suspended in dimethyl sulfoxide (DMSO), and then diluted to be subjected to biological analyses. Sterile filter paper discs (5 mm) were impregnated with volumes corresponding to 0.4, 1 and 2 µL of the essential oil and placed on the plates. These last were left for 30 min at room temperature under sterile conditions before their incubation at 37 °C for 24 h; the diameter of the clear zone shown on plates (inhibition halo zone) was accurately measured (“Extra steel Caliper mod 0289”, mm/inch reading scale, precision 0.05 mm, Mario De Maio, Milan, Italy). A disc treated with DMSO alone served as the negative control. Tetracycline (7 µg/disc; Sigma Aldrich, Milano, Italy) was used as reference drugs. The experiments were performed in triplicate and averaged.

### 4.7. Minimum Inhibitory Concentration (MIC)

A modified version of the resazurin microtitre-plate assay [41] was used to evaluate the minimal inhibitory concentration of the *L. nobilis* essential oil and 1,8-cineole. Briefly, different volumes of the test material—prepared as described above—were pipetted in a multi-well with different volumes of Muller-Hinton broth (Sigma Aldrich, Milano, Italy). Two fold serial dilutions were performed such that each well had 50 μL of the test material in serially descending concentrations. 30 μL of 3.3 × strength isosensitised broth (Sigma Aldrich) and 5 μL of resazurin indicator solution (previously prepared by dissolving 270 mg tablet purchased from Sigma Aldrich, in 40 mL of sterile distilled water) were added in each well, to reach a final volume/well of 240 µL. Finally, 10 μL of bacterial suspension was added to each well to achieve a concentration of approx. 5 × 10^5^ cfu/mL. Ciprofloxacin (3 µM in DMSO, Sigma Aldrich) and DMSO were used as positive and negative controls, respectively. Plates were prepared in triplicate, wrapped with cling film to avoid the bacterial dehydration, and incubated at 37 °C for 24 h. The lowest concentration at which a colour change (visually assessed from blu/dark purple to pink, or colorless) occurred indicated the MIC value.

### 4.8. Antifungal Activity

The possible antifungal activity of the essential oil and 1,8-cineole was tested against four fungal tester strains of agro-food interest, Aspergillus versicolor DSM 1943, Penicillium expansum DSM 1994, Penicillium citrinum DSMZ 1179, and Aspergillus niger DSM 1957 following the method of Marrufo and co-workers [42]. The strains were purchased from DSMZ. They were grown in potato dextrose broth (Sigma Aldrich, Milano, Italy) at 28 °C. A cell suspension of fungi was prepared in sterile distilled water, adjusted to contain approximately 10^6^ CFU/mL, and plated onto potato dextrose agar. After 30 min under sterile conditions, the inoculated plates were spotted with different amount of the samples, previously diluted 1:10 (*v*/*v*) in DMSO. After 20 min under sterile conditions at room temperature, plates were incubated at 28 °C for 48–72 h. When the mycelium of fungi reached the edges of the control plate (negative control without the samples added), the diameter of the clear zone shown on plates (inhibition halo zone) was accurately measured (“Extra steel Caliper mod 0289”, mm/inch reading scale, precision 0.05 mm, Mario De Maio, Milano, Italy); DMSO was used as negative control (10 μL/paper disc). The antifungal activity was expressed in mm. Samples were tested in triplicate and the results are expressed as mean ± standard deviation.

### 4.9. Cell Cultures

Human neuroblastoma (SH-SY5Y) cancer cells were cultured in Roswell Park Memorial Institute Medium (RPMI) supplemented with 1% l-glutamine, 10% heat-inactivated fetal bovine serum (FBS), 1% penicillin/streptomycin (all from Sigma Aldrich) at 37 °C in an atmosphere of 95% O_2_ and 5% CO_2_.

### 4.10. MTT Assay

Cells were plated (7 × 103) in 96-well culture plates in 150 µL of culture medium and incubated at 37 °C in humidified atmosphere of 95% O_2_ and 5% CO_2_. The day after, a 150 µL aliquot of serial dilutions of 1,8- cineole and the essential oil (1600–50 µg/mL) was added to the cells and incubated for 24 h. DMSO alone was used as control. Cell viability was assessed through MTT (3-(4,5-dimethylthiazol-2-yl)-2,5-diphenyl tetrazolium bromide) assay. Briefly, 30 µL of MTT (5 mg/mL) was added and the cells incubated for additional 3 h. Thereafter, cells were lysed and the dark blue crystals solubilized with 30 µL of a solution containing 50%, *v*/*v*, *N*,*N*-dimethylformamide, 20%, *w*/*v*, SDS with an adjusted pH of 4.5. The optical density (OD) of each well was measured with a microplate spectrophotometer (Thermo Scientific Multiskan GO) equipped with a 520 nm filter. Cell viability in response to treatment was calculated as a percentage of control cells treated with DMSO at the final concentration 0.1% viable cells = (100 × OD treated cells)/OD control cells [43].

### 4.11. Extraction Proteins and Western Blotting

Cells were treated with different concentrations (400–50 µg/mL) of the 1,8-cineole and essential oil, and after 24 h, they were collected and lysed using Laemmli buffer to extract total proteins. For Western blot analysis, an aliquot of total protein was run on 8% SDS-PAGE gels and transferred to nitrocellulose. Nitrocellulose blots were blocked with 10% non-fat dry milk in Tris buffer saline 0.1% Tween-20 over night at 4 °C. After blocking, blots were incubated with antibodies raised against ADCY1 (Santa Cruz Biotechnology, Santa Cruz, CA, USA), phosphorylated p44/42 MAP kinase (ERK), or total phosphorylated p44/42 MAP kinase (ERK) for 3 h at room temperature. Immunoreactivity was detected by sequential incubation with horseradish peroxidase-conjugated secondary antibody (Amersham Biosciences, Pittsburgh, PA, USA) and enhanced chemiluminescence reagents (ImmunoCruz, Santa Cruz Biotechnology, Santa Cruz, CA, USA) [44].

### 4.12. Statistical Analysis

All experiments were carried out in triplicate. Data of each experiment were statistically analyzed using GraphPad Prism 6.0 software (GraphPad Software Inc., San Diego, CA, USA) followed by comparison of means (two-way ANOVA) using Dunnett’s multiple comparisons test, at the significance level of *p* < 0.05.

## Figures and Tables

**Table 1:** Chemical composition of the essential oil (EO) isolated from the leaves of *L. nobilis.*

No.	Compound	%	Ri ^a^	Ri ^b^	Identification ^c^
**1**	Methyl pentanoate	0.1	850	828	1,2
**2**	Ethyl isovalerate	0.1	853	858	1,2
**3**	α-Thujene	0.7	916	930	1,2
**4**	α-Pinene	5.8	922	939	1,2,3
**5**	Camphene	0.8	935	954	1,2
**6**	Sabinene	12.2	962	975	1,2
**7**	β-Pinene	1.4	980	979	1,2,3
**8**	α-Phellandrene	0.5	991	1002	1,2,3
**9**	δ-2-Carene	0.4	997	1002	1,2
**10**	α-Terpinene	0.6	1004	1017	1,2,3
**11**	*o*-Cymene	0.3	1013	1026	1,2
**12**	1,8-Cineole	31.9	1016	1031	1,2,3
**13**	(*Z*)-β-Ocimene	0.2	1028	1037	1,2
**14**	(*E*)-β-Ocimene	0.2	1038	1050	1,2
**15**	γ-Terpinene	1.0	1048	1059	1,2,3
**16**	*cis*-Sabinene hydrate	0.3	1057	1070	1,2
**17**	ρ-Mentha-3,8-diene	0.5	1077	1072	1,2
**18**	*trans*-Sabinene hydrate	10.2	1093	1098	1,2
**19**	Linalool	0.1	1096	1096	1,2,3
**20**	*exo*-Fenchol	0.1	1111	1121	1,2
**21**	*allo*-Ocimene	0.2	1118	1132	1,2
**22**	*trans*-Sabinol	0.2	1128	1142	1,2
**23**	Camphor	0.2	1133	1146	1,2,3
**24**	β-Pinene oxide	0.1	1147	1159	1,2
**25**	Isoborneol	0.5	1155	1160	1,2
**26**	*iso*-Isopulegol	0.6	1157	1159	1,2
**27**	*neoiso*-Isopulegol	2.5	1165	1171	1,2
**28**	α-Terpineol	3.3	1180	1188	1,2,3
**29**	*cis*-Carveol	0.2	1219	1229	1,2
**30**	*cis*-*p*-Mentha-1(7),8-dien-2-ol	0.1	1232	1230	1,2
**31**	*trans*-Sabinene hydrate acetate	0.7	1246	1256	1,2
**32**	2-(1*E*)-Propenyl-phenol	0.1	1265	1267	1,2
**33**	*neo*-3-Thujanol acetate	0.4	1275	1276	1,2
**34**	α-Terpinen-7-al	0.3	1284	1285	1,2
**35**	*iso*-Verbanol acetate	0.3	1306	1309	1,2
**36**	α-Terpinyl acetate	5.9	1340	1349	1,2
**37**	Eugenol	1.6	1347	1359	1,2,3
**38**	Cyclosativene	0.1	1360	1371	1,2
**39**	Longicyclene	0.2	1373	1374	1,2
**40**	β-Elemene	0.4	1381	1390	1,2
**41**	Methyl-eugenol	3.3	1394	1403	1,2,3
**42**	β-Funebrene	0.5	1408	1414	1,2
**43**	*cis*-Thujopsene	0.2	1427	1431	1,2
**44**	Spirolepechinene	0.1	1445	1451	1,2
**45**	*allo*-Aromadendrene	0.1	1449	1460	1,2,3
**46**	γ-Himachalene	0.1	1474	1482	1,2
**47**	*a*-Amorphene	0.1	1483	1484	1,2
**48**	δ-Amorphene	0.1	1502	1512	1,2
**49**	δ-Cadinene	0.2	1512	1523	1,2
**50**	Elemicin	0.5	1546	1557	1,2
**51**	Spathulenol	0.4	1563	1578	1,2,3
**52**	Caryophyllene oxide	0.3	1572	1583	1,2,3
**53**	Thujopsan-2-α-ol	0.1	1580	1587	1,2
**54**	Viridiflorol	0.2	1591	1592	1,2
**55**	Eremoligenol	0.1	1630	1631	1,2
	**Total**	**91.6**			
	Monoterpenes hydrocarbons	34.0			
	Oxygenated monoterpenes	48.6			
	Sesquiterpene hydrocarbons	3.2			
	Oxygenated sesquiterpenes	0.2			
	Phenolic compounds	5.6			

^a^ Linear retention index on a HP-5MS column; ^b^ Linear retention index on a HP Innowax column; ^c^ Identification method: 1 = linear retention index; 2 = identification based on the comparison of mass spectra; 3 = Co-injection with standard compounds.

**Table 2:** Antimicrobial activity the EO of *Laurus nobilis* and 1,8-cineole.

Bacterial Strains	Inhibition Diameter (mm)						
	*Laurus nobilis* Essential Oil			1,8-cineole			Tetracycline
	0.4 µL/mL	1 µL/mL	2 µL/mL	0.4 µL/mL	1 µL/mL	2 µL/mL	7 µg/mL
*B.cereus* 4313	8.66 ± 1.54 ^b^	14.66 ± 0.57 ^c^	18 ± 0 ^e^	5.66 ± 1.54 ^e^	12 ± 2.64 ^c^	14.66 ± 0.57 ^d^	10.33 ± 0.57 ^a^
*B. cereus* 4384	7.66 ± 1.54 ^b^	12 ± 2.64 ^d^	15.66 ± 0.57 ^e^	5.66 ± 1.54 ^c^	11.66 ± 1.54 ^c^	14.66 ± 0.57 ^e^	8.67 ± 1.67 ^a^
*S. aureus*	8.33 ± 0.57 ^c^	11.66 ± 1.54 ^a^	13.33 ± 1.54 ^b^	0 ± ^e^	7.66 ± 1.54 ^d^	12 ± 1.54 ^a^	11.33 ± 0.57 ^a^
*E. coli*	6.33 ± 0.57 ^e^	12 ± 0 ^a^	16 ± 2 ^e^	0 ± ^e^	0 ± ^e^	5.66 ± 1.54 ^e^	12.70 ± 1.67 ^a^
*P. aeruginosa*	8.33 ± 1.54 ^b^	12 ± 1.73 ^d^	15.33 ± 0.57 ^e^	0 ± ^e^	7.66 ± 1.54 ^c^	12 ± 1.73 ^d^	9.67 ± 0.57 ^a^

Data are expressed in mm. Results are shown as the mean ± SD (*n* = 3). Means followed by different letters in each column differ significantly to Dunnett’s multiple comparisons test, at the significance level of *p* < 0.05. ^e^: *p* < 0.0001; ^d^: *p* < 0.001; ^c^: *p* < 0.001; ^b^: *p* < 0.005; ^a^: *p* < 0.05 vs. tetracycline (7 µg).

**Table 3:** Minimal inhibitory concentration (MIC, µL) of the EO of *Laurus nobilis* and of 1,8-cineole.

Microorganism	MIC (µL/mL)	
	*Laurus nobilis*	1,8-cineole
*Bacillus cereus 4313*	0.2	0.2
*Bacillus cereus 4384*	0.2	0.4
*Staphylococcus aureus*	0.4	1
*Escherichia coli*	0.8	1.5
*Pseudomonas aeruginosa*	0.4	1

**Table 4:** Antifungal activity of *L. nobilis* essential oil and of 1,8-cineole.

	*A. niger*	*A. versicolor*	*P. citrinum*	*P. expansum*
*Laurus nobilis* EO				
0.4 µL	2 ± 0	-	2.33 ± 0.57	8 ± 1.73
1 µL	4.33 ± 1.52	5.66 ± 1.54	4.66 ± 0.57	9.33 ± 2.08
2 µL	6 ± 1	7.66 ± 1.54	5.66 ± 1.54	9.66 ± 0.57
1,8-cineole				
2 µL	-	-	-	-
4 µL	-	-	-	-
8 µL	5.66 ± 1.54	7.66 ± 1.54	5.66 ± 1.54	9.66 ± 0.57

Data are expressed in mm. Results are shown as mean ± standard deviation (SD) of the inhibition zone (*n* = 3).
